# Adverse Trends in Ischemic Heart Disease Mortality among Young New Yorkers, Particularly Young Black Women

**DOI:** 10.1371/journal.pone.0149015

**Published:** 2016-02-16

**Authors:** Nathaniel R. Smilowitz, Gil A. Maduro, Iryna V. Lobach, Yu Chen, Harmony R. Reynolds

**Affiliations:** 1 Cardiovascular Clinical Research Center, NYU School of Medicine, New York, NY, United States of America; 2 New York City Department of Health and Mental Health, New York, NY, United States of America; 3 Department of Biostatistics, NYU School of Medicine, New York, NY, United States of America; 4 Department of Population Health, NYU School of Medicine, New York, NY, United States of America; University of Bologna, ITALY

## Abstract

**Background:**

Ischemic heart disease (IHD) mortality has been on the decline in the United States for decades. However, declines in IHD mortality have been slower in certain groups, including young women and black individuals.

**Hypothesis:**

Trends in IHD vary by age, sex, and race in New York City (NYC). Young female minorities are a vulnerable group that may warrant renewed efforts to reduce IHD.

**Methods:**

IHD mortality trends were assessed in NYC 1980–2008. NYC Vital Statistics data were obtained for analysis. Age-specific IHD mortality rates and confidence bounds were estimated. Trends in IHD mortality were compared by age and race/ethnicity using linear regression of log-transformed mortality rates. Rates and trends in IHD mortality rates were compared between subgroups defined by age, sex and race/ethnicity.

**Results:**

The decline in IHD mortality rates slowed in 1999 among individuals aged 35–54 years but not ≥55. IHD mortality rates were higher among young men than women age 35–54, but annual declines in IHD mortality were slower for women. Black women age 35–54 had higher IHD mortality rates and slower declines in IHD mortality than women of other race/ethnicity groups. IHD mortality trends were similar in black and white men age 35–54.

**Conclusions:**

The decline in IHD mortality rates has slowed in recent years among younger, but not older, individuals in NYC. There was an association between sex and race/ethnicity on IHD mortality rates and trends. Young black women may benefit from targeted medical and public health interventions to reduce IHD mortality.

## Introduction

One of the great achievements of 20^th^ century medicine has been the reduction in mortality from cardiovascular disease. This progress is attributed to advances in medical, percutaneous, and surgical therapies for ischemic heart disease and modification of established risk factors such as hyperlipidemia, hypertension and smoking.[1]

Unfortunately, in recent years improvements have not been universal. The rate of death from ischemic heart disease (IHD) in the United States did not decline among women aged 35–44 from 2000–2002, in contrast to men aged 35–44 and older individuals of both sexes.[2] The reasons for stagnant or slower declines of IHD mortality rates in young women are not known. This adverse trend may represent increasing incidence of IHD including risk factors for IHD in this population, worsening outcomes among young women who do develop IHD, disparities in care, incorrect assignment of IHD as the cause of death, and/or combinations of the above. The impact of race/ethnicity on adverse trends in IHD mortality among young adults has not been fully described.

The population of New York City (NYC), the largest city in the United States, has a higher proportion of women and a lower proportion of elderly, rendering it desirable for in-depth analyses of trends in IHD mortality among young individuals without confounding by region or urbanization.[3] We set out to investigate trends in mortality rates by sex, age, and race/ethnicity in a single, large urban population.

## Methods

The study was approved by the New York University School of Medicine Institutional Review Board with exemption from review. NYC vital statistics data were used to prepare mortality rates from 1980 to 2008. Causes of death, documented on the death certificate, were coded according to ICD-9 codes for years 1980–1998 and ICD-10 codes for years 1999–2008. The codes corresponding to IHD are 410–414 (ICD-9) and I20-25 (ICD-10). Age, sex, and race/ethnicity of decedents, typically provided by next of kin and documented on the death certificate by funeral directors, were obtained in aggregate without personal identifiers. Race/ethnicity categories were available for the period from 1990 to 2008 and were defined as non-Hispanic (NH) white (further referred to here as white), NH black (further referred to here as black), Hispanic, and Asian/Pacific Islander. Age-specific population mortality rates were estimated per 100,000 for each subgroup based on US Census data. Mortality trend curves for IHD were constructed by age, sex, and race/ethnicity and displayed on absolute and logarithmic scales. Age groups of 35–54 years and ≥55 were selected based on prior literature and ongoing studies.[2, 4] Trends in mortality rates for women age 35–54 were compared to younger men age 35–54, as well as to older men and women. Nonparametric analysis identified significant points of change in mortality trends between 1997 and 2000 for all age and sex subgroups. The most frequently identified point of change (1999) was selected for temporal sub-analyses.

### Statistical Analysis

Trends in absolute and log-transformed mortality rates were explored using linear regression with the speed of change estimated by the regression line slope. We calculated annual proportional decline, i.e., the annual percent of rate reduction, for each underlying diagnosis and each comparison group based on the slope estimates from linear regression of log-transformed mortality rates. Differences in slopes were tested using two-sided t-tests of differences in slopes on the log scale. To adjust for multiple testing, we used a conservative Bonferroni adjustment. To examine deviations in the observed relationship between mortality rates and time from the linear regression line, we considered piece-wise linear regression. The piece-wise linear regression was fit within visually defined intervals. Regressions corresponding to the smallest deviation of the observed relationship from the linear fit within each interval were reported. Homoscedasticity and independence of the residuals assumptions were checked. To further explore nonlinearity and change-points in the relationship between mortality rates and time, we employed nonparametric, resampling-based technique available in Joinpoint 3.5.1 software.[5]

Four-way and all possible 2-way comparisons adjusted using Bonferroni multiple testing correction were performed to compare slopes of log-transformed mortality curves for each underlying cause category using the following comparisons: 1) women vs. men aged 35–54 by race/ethnicity; 2) women aged 35–54 by race/ethnicity; 3) women aged 35–54 vs. women ≥55 by race/ethnicity 4) women aged ≥55 by race/ethnicity, and 5) women vs. men aged ≥55 by race/ethnicity.

## Results

### Ischemic heart disease mortality trends by age and sex

**Fig 1:**
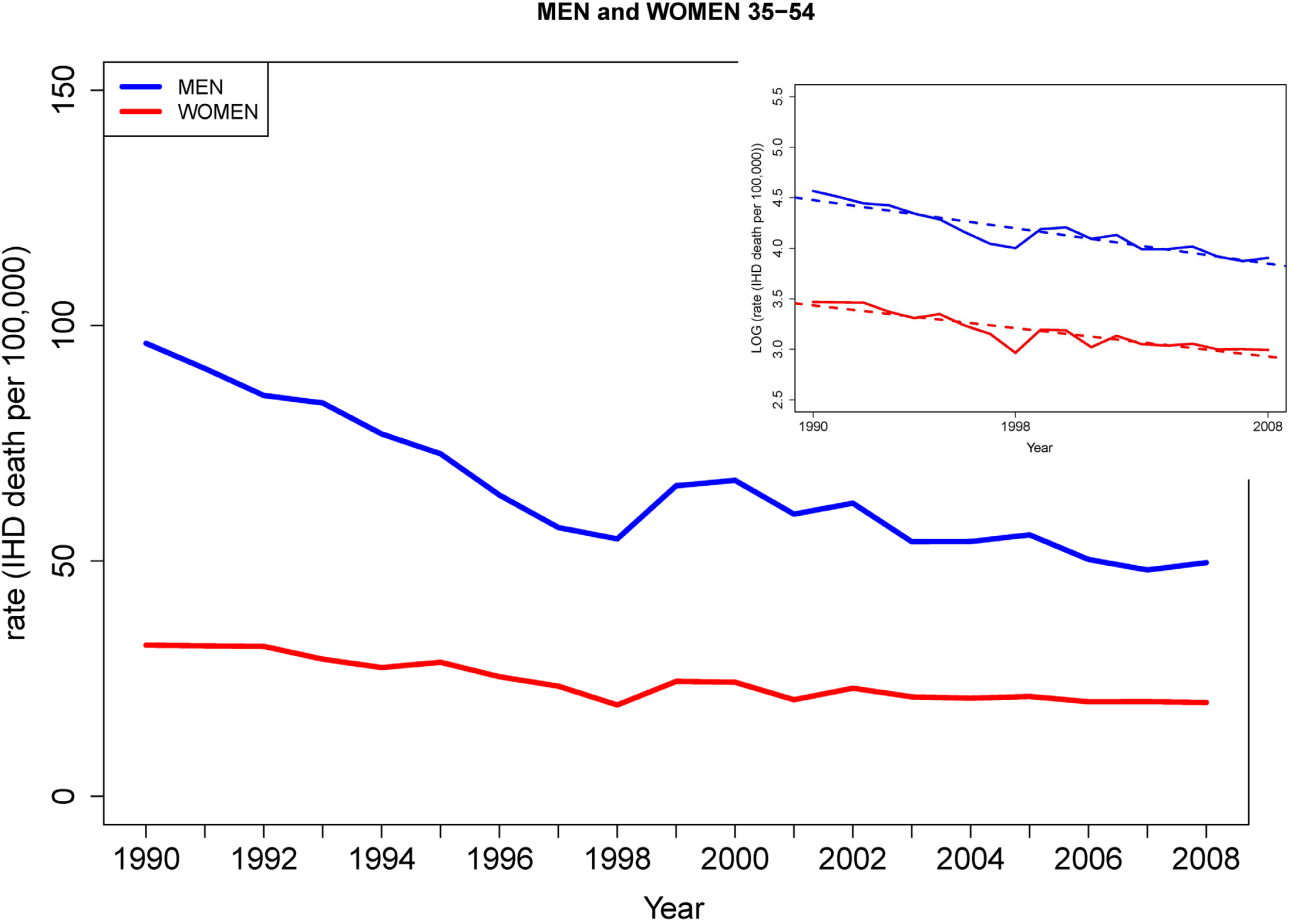
IHD mortality trends in men and women age 35–54 (1980–2008). Absolute and log transformed rates are shown (inset panel). Men 35–54 have higher rates of IHD mortality and faster decline in IHD mortality than women.

**Fig 2:**
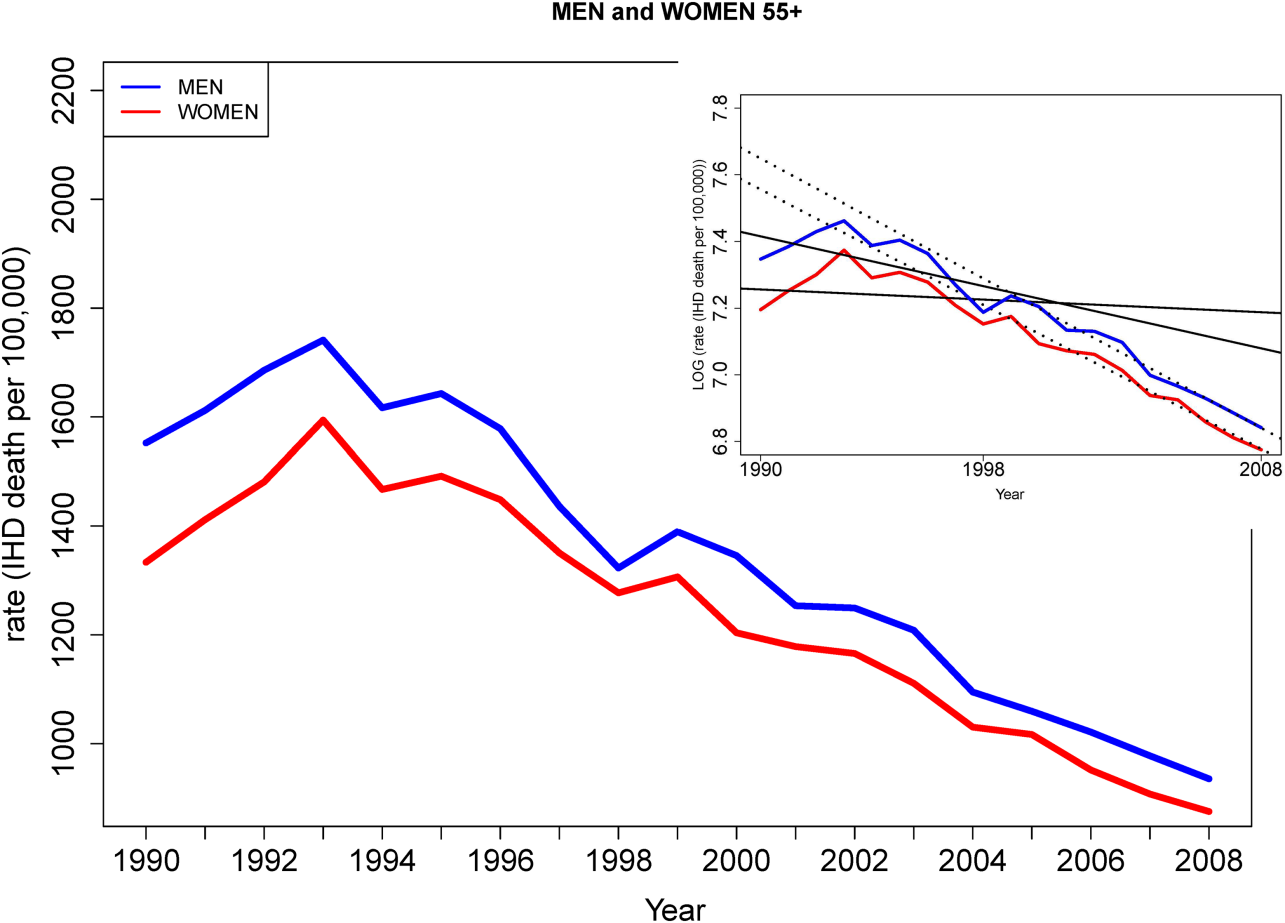
IHD mortality trends in men and women age ≥55 (1980–2008). Absolute and log transformed rates are shown (inset panel). IHD mortality trends in men and women 55+ show two distinct rates of decline from 1980–1999. Trends in IHD mortality declines did not differ by sex from 1999–2008.

IHD mortality decreased for women and men of all age groups from 1980 to 2008. Absolute declines in IHD mortality were greater for the ≥55 age subgroup than the subgroup aged 35–54 during the study period Figs 1 & 2. Annual percent declines in IHD mortality rates slowed among younger men and women age 35–54 from 1999–2008 Fig 1 compared with annual percent declines from 1980–1998 (3.4% vs. 6.3%, Table 1) but not among men and women aged ≥55 Fig 2, who experienced accelerated declines in IHD mortality rate in the later study period (4.4% from 1999–2008 vs. 1.0% from 1990–1998, Table 1). Age-specific IHD mortality rates from 1980–2008 were as follows: A decline from 52.8 to 19.9 per 100,000 for women aged 35–54, 179.8 to 49.6 for men aged 35–54, 1517.0 to 875.5 for women aged ≥55 and 2049.1 to 935.5 for men aged ≥55. See Tables 2 and 3 for annual mortality data.

**Table 1:** Annual proportional decline in ischemic heart disease mortality by age and sex, NYC 1980–2008.

	Population Subgroup								
Underlying cause of Death	35–54	≥55	P	Women 35–54	Men 35–54	P	Women ≥55	Men ≥55	P
**Ischemic Heart Disease**	4.7%	1.9%	**<0.001**	4.0%	4.9%	0.01	1.6%	2.5%	**0.002**
1980–1998	6.3%	1.0%	**<0.001**	5.6%	6.8%	0.01	0.4%	1.9%	0.004
1999–2008	3.4%	4.4%	0.44	2.3%	4.0%	0.14	4.3%	4.5%	0.54
P for 1980–1998 vs. 1999–2008	0.007	0.006		**<0.001**	0.004		**<0.001**	**<0.001**	

**Table 2:** Ischemic heart disease (IHD) mortality rates (per 100,000) by age and race/ethnicity in NYC for women.

	Female IHD Mortality											
Year	n =		All		White NH		Black NH		Hispanic		Asian	
	35–54	55+	35–54	55+	35–54	55+	35–54	55+	35–54	55+	35–54	55+
**1980**	852,158	1,002,544	52.8	1517.0	-	-	-	-	-	-	-	-
**1981**	850,993	1,001,276	58.9	1477.2	-	-	-	-	-	-	-	-
**1982**	864,749	1,000,779	57.1	1526.4	-	-	-	-	-	-	-	-
**1983**	880,935	1,003,487	56.6	1530.2	-	-	-	-	-	-	-	-
**1984**	897,227	1,002,913	49.4	1539.5	-	-	-	-	-	-	-	-
**1985**	911,379	999,586	46.6	1468.9	-	-	-	-	-	-	-	-
**1986**	930,427	996,077	35.6	1349.2	-	-	-	-	-	-	-	-
**1987**	947,086	988,263	37.4	1263.6	-	-	-	-	-	-	-	-
**1988**	964,749	973,914	33.6	1291.0	-	-	-	-	-	-	-	-
**1989**	979,990	957,120	33.8	1273.7	-	-	-	-	-	-	-	-
**1990**	996,481	943,676	32.1	1333.4	28.2	1566.8	51.6	1035.4	21.1	516.7	8.7	887.9
**1991**	1,010,284	933,690	32.0	1411.6	28.9	1651.9	42.2	1079.5	24.4	581.6	10.7	975.2
**1992**	1,026,480	926,482	31.9	1481.1	27.0	1758.3	49.6	1153.2	19.4	587.0	7.4	568.5
**1993**	1,047,073	922,126	29.1	1594.1	23.1	1925.5	47.9	1190.5	16.5	594.8	9.1	390.0
**1994**	1,067,714	918,647	27.3	1467.3	24.5	1901.4	44.8	1141.5	19.5	583.6	3.2	440.5
**1995**	1,088,642	917,554	28.5	1491.6	24.9	1970.3	46.6	1139.0	21.2	602.9	9.9	478.7
**1996**	1,114,137	915,713	25.4	1448.5	23.0	1922.4	44.7	1125.6	14.8	597.0	6.4	519.0
**1997**	1,137,070	918,183	23.4	1350.5	23.6	1820.0	37.3	1037.5	15.9	575.7	4.3	447.5
**1998**	1,160,078	925,181	19.4	1277.3	18.2	1740.8	31.3	949.6	12.4	562.3	8.1	422.5
**1999**	1,179,899	942,551	24.4	1306.6	25.9	1739.4	38.1	1027.6	14.8	654.1	3.1	427.4
**2000**	1,197,069	959,269	24.2	1203.8	19.4	1634.0	45.6	977.3	15.7	584.5	6.1	407.2
**2001**	1,214,189	975,744	20.5	1178.5	20.7	1622.5	32.4	955.8	11.8	552.7	7.9	440.0
**2002**	1,223,532	993,336	23.0	1165.9	20.7	1630.4	41.9	957.7	14.7	525.0	4.2	424.0
**2003**	1,230,439	1,011,003	21.1	1111.1	23.1	1477.2	31.1	923.1	14.1	568.3	4.7	413.4
**2004**	1,233,950	1,026,823	20.8	1030.5	18.2	1387.9	37.5	887.4	13.9	518.3	4.7	407.0
**2005**	1,239,738	1,050,151	21.2	1017.0	18.9	1409.7	38.1	863.6	14.5	528.8	7.1	391.6
**2006**	1,249,760	1,061,828	20.1	951.8	14.1	1315.9	40.6	832.7	15.3	506.7	5.7	349.8
**2007**	1,267,877	1,079,080	20.1	907.5	14.3	1249.0	39.8	811.7	17.5	487.9	3.1	363.9
**2008**	1,278,542	1,105,479	19.9	875.5	17.3	1198.6	37.8	783.8	13.2	496.6	4.8	364.2

**Table 3:** Ischemic heart disease (IHD) mortality rates (per 100,000) by age and race/ethnicity in NYC for men.

	Male IHD Mortality											
Year	n =		All		White NH		Black NH		Hispanic		Asian	
	35–54	55+	35–54	55+	35–54	55+	35–54	55+	35–54	55+	35–54	55+
**1980**	733,407	687,624	179.8	2049.1	-	-	-	-	-	-	-	-
**1981**	734,431	684,392	175.9	1978.3	-	-	-	-	-	-	-	-
**1982**	748,978	682,406	168.9	2049.8	-	-	-	-	-	-	-	-
**1983**	765,238	683,369	150.5	2001.1	-	-	-	-	-	-	-	-
**1984**	781,406	681,853	149.9	1930.9	-	-	-	-	-	-	-	-
**1985**	795,743	679,262	138.4	1849.9	-	-	-	-	-	-	-	-
**1986**	814,880	675,021	122.7	1703.2	-	-	-	-	-	-	-	-
**1987**	832,092	668,412	120.7	1597.8	-	-	-	-	-	-	-	-
**1988**	849,757	657,626	108.5	1552.7	-	-	-	-	-	-	-	-
**1989**	865,317	646,124	99.8	1511.2	-	-	-	-	-	-	-	-
**1990**	882,267	637,038	96.2	1552.2	98.4	1758.5	120.2	1261.6	68.5	702.5	35.2	1065.3
**1991**	896,854	632,222	90.9	1611.9	93.3	1836.4	115.7	1230.4	56.1	746.1	53.7	1155.1
**1992**	913,710	629,888	85.1	1685.7	89.2	1971.7	97.2	1311.4	56.8	724.8	44.2	724.5
**1993**	934,781	629,455	83.5	1741.2	94.0	2051.6	97.8	1361.2	53.6	729.8	24.9	620.9
**1994**	954,812	628,706	77.0	1616.8	88.1	2039.7	101.2	1200.8	45.3	804.4	34.4	611.2
**1995**	975,776	630,387	72.8	1643.0	84.8	2105.2	87.7	1304.2	46.2	735.9	43.9	620.4
**1996**	1,000,189	631,735	64.0	1578.4	81.0	2014.8	65.9	1278.3	38.0	783.5	36.8	640.1
**1997**	1,022,882	636,008	57.1	1436.3	67.0	1845.8	60.4	1172.7	42.2	730.0	34.2	583.1
**1998**	1,046,140	649,105	54.7	1322.9	70.4	1711.1	59.8	1048.2	33.0	659.0	29.8	605.4
**1999**	1,068,657	658,447	66.0	1389.6	76.6	1760.6	79.0	1150.2	48.7	742.8	25.6	641.3
**2000**	1,087,695	668,374	67.1	1345.7	86.6	1731.8	77.7	1179.6	40.5	714.4	31.9	563.3
**2001**	1,109,421	681,960	59.9	1253.6	75.3	1627.8	70.8	1091.4	38.4	713.4	32.6	529.4
**2002**	1,122,562	696,723	62.3	1249.4	80.0	1621.2	75.9	1111.9	37.6	704.3	31.4	530.2
**2003**	1,133,506	711,912	54.1	1208.6	67.2	1516.9	64.7	1061.6	33.1	696.2	28.5	520.2
**2004**	1,143,543	723,967	54.1	1094.9	55.6	1402.8	79.7	979.1	38.5	657.7	30.5	463.5
**2005**	1,155,788	742,757	55.5	1059.7	58.6	1370.8	70.6	1013.7	45.1	611.1	37.8	478.7
**2006**	1,172,581	763,423	50.3	1021.5	58.3	1325.4	64.6	943.8	36.8	635.1	27.5	457.0
**2007**	1,194,040	781,215	48.1	977.8	48.2	1275.3	70.3	901.8	34.4	618.4	32.9	447.2
**2008**	1,208,639	804,987	49.6	935.5	52.8	1206.0	72.9	896.5	40.2	582.2	22.3	461.6

Note that mortality data for race/ethnicity were not available before 1990. Population n refers to the population in NYC for women or men in the labeled age category by year.

Among younger individuals aged 35–54, men displayed higher absolute rates of IHD mortality than women. The annual percent declines in IHD mortality were more favorable for men than for women during the period from 1980–1999 (6.8% vs. 5.6% per year, p = 0.01; see Table 1 and Fig 1 and were similar for both sexes from 1999–2008.

Men aged ≥55 displayed higher IHD mortality rates than women ≥55 at all time-points of the study, but sex differences narrowed due to a faster decline in IHD mortality in men from 1980–1998 (1.9% vs. 0.4% per year, p = 0.004; see Table 1 and Fig 2. Annual percent declines in IHD mortality were similar for men and women in the later part of the study period.

### Ischemic heart disease mortality trends by race/ethnicity

**Fig 3:**
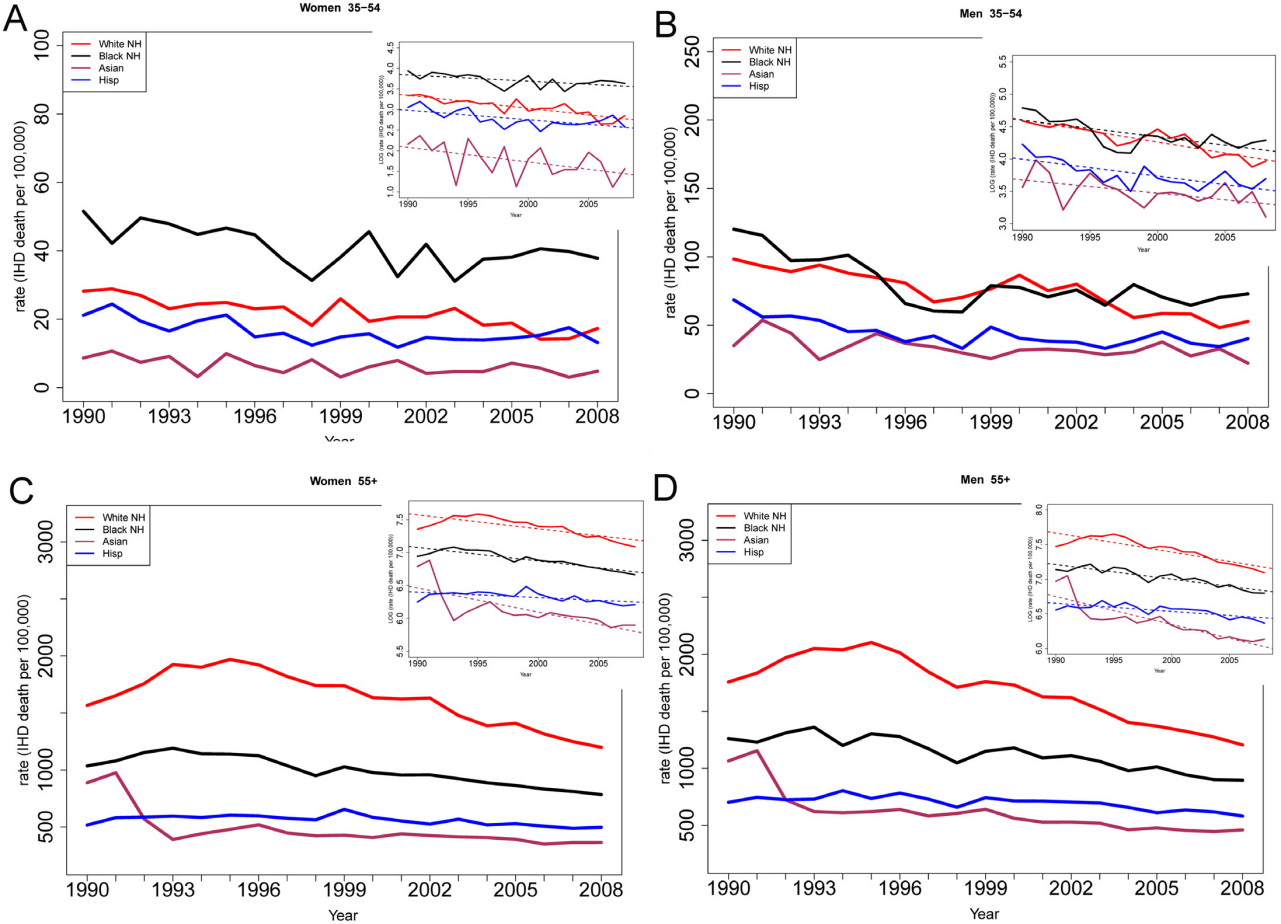
IHD mortality trends in women age 35–54 by race/ethnicity (1990–2008). **a:** Black women 35–54 had higher rates of IHD mortality and slower decline in IHD mortality than white women. **b:** *IHD mortality trends in men age 35–54 by race/ethnicity (1990–2008)*. Among men 35–54, rates of IHD mortality decline did not differ by ethnicity. **c:** *IHD mortality trends in women age ≥55 by race/ethnicity (1990–2008)*. Among women 55+, the rate of decline in IHD morality in Asians was greater than the rate of decline in whites or blacks. **d:** *IHD mortality trends in men age ≥55 by race/ethnicity (1990–2008)*. Among men 55+, the rate of decline in IHD morality in Hispanics was slower than the rate of decline in whites or blacks.

IHD mortality decreased between 1990–2008 for all race/ethnicity categories. Among women aged 35–54, the absolute IHD mortality rate was higher among blacks than other race/ethnicity groups Fig 3a. Younger black women also experienced slower declines in IHD mortality than other race/ethnicity groups (see Table 4). Among men 35–54, absolute IHD mortality rates were higher among black and whites than Hispanic and Asian men Fig 3b. The annual percent decline in IHD mortality among men aged 35–54 was similar for all race/ethnicity groups during the study period (Table 4).

**Table 4:** Annual proportional decline in ischemic heart disease mortality by age, sex and race/ethnicity, NYC 1990–2008.

	Ischemic Heart Disease	p (vs. white)
**Women 35–54**		
White NH	3.2%	-
Hispanic	2.3%	0.81
Black NH	1.5%	0.03
Asian	3.5%	0.28
**Men 35–54**		
White NH	3.4%	-
Hispanic	2.6%	0.13
Black NH	2.6%	0.28
Asian	2.0%	0.27
**Women 55+**		
White NH	2.1%	-
Hispanic	0.8%	0.09†
Black NH	2.1%	0.89
Asian	3.7%	0.01†
**Men 55+**		
White NH	2.7%	-
Hispanic	4.0%	0.06†
Black NH	2.1%	0.13
Asian	1.2%	<0.001†

^†^ p<0.01 with Black in same age—sex category

Among individuals age ≥55, black women and men had lower rates of mortality than whites (but higher than Hispanics and Asians) Fig 3c and 3d. Asian women experienced more rapid declines in IHD mortality over the study period as compared to women ≥55 in other race/ethnicity groups, while Hispanic women experienced slower declines (Table 4). Conversely, among men aged ≥55, Asians experienced a slower decline in IHD mortality and Hispanics a faster decline as compared to white and black non-Hispanic men.

## Discussion

We identified a slowing rate of decline in ischemic heart disease mortality among young (35–54) but not older (≥55) New Yorkers in recent years. We also identified a striking relationship between age, sex, and race/ethnicity and IHD mortality rates in NYC, with slower declines of IHD mortality in young black women than young women of other race/ethnic groups. Trends in IHD mortality in NYC are of particular interest because IHD mortality in NY State is among the highest in the nation and IHD mortality is higher in large metropolitan areas.[6] In this analysis of NYC Vital Statistics data, IHD mortality rates among young individuals were higher than those reported nationally. These findings complement data from large cohort studies that demonstrate wide racial differences in fatal IHD rates and steeper declines in IHD mortality for whites than for blacks over the past two decades, adding analysis of trends by race and sex among younger individuals.[7, 8]

### Potential explanations for adverse trends among younger individuals

Ischemic heart disease is the leading cause of cardiovascular death among young individuals in the United States, accounting for approximately half of all cardiovascular deaths.[9] Adverse trends in mortality among younger individuals are of particular public health concern because of the large number of years of potential life lost. Slowing of the IHD mortality decline among young individuals since 1999 was most likely due to an increase in the incidence of IHD among younger individuals. NYC Community Health Survey data show that the prevalence of IHD risk factors obesity and diabetes in NYC increased by nearly 20% from 2002 to 2004, a rate significantly faster than the national average.[10] Although obesity rates increased in both younger and older adults in NYC, epidemiologic studies demonstrate stronger associations between cardiovascular risk factors and mortality in younger individuals.[10, 11] An increase in the case fatality rate for IHD during the study period could also theoretically explain the slowing of the IHD mortality decline. Although national data for case fatality rate show a decrease in that time period for both men and women, analysis by age group was not performed.^8^

### Sex differences in ischemic heart disease mortality trends

Among younger individuals, women experienced a slower annual proportional decline in IHD mortality than did men. Declines in IHD mortality favoring men have also been reported in Finnish registries.[4] Although IHD mortality among men remains higher than that for women, the sex difference in the downward trend of IHD mortality is of particular concern. These trends may reflect a lack of emphasis on prevention and early detection of IHD among younger women due to lower absolute risks of IHD or could reflect sex differences in treatment and outcomes.[12] Women age ≤55 with acute coronary syndromes have the highest risk of being improperly sent home from the emergency department, and are less likely to receive coronary revascularization than men.[13–16] Case fatality rates for myocardial infarction are higher for young women than young men, with no sex difference among older patients.[16–18] This sex discrepancy persists even after adjustment for demographics, medical history, treatment, and complications. Shifting demographics and the migration of higher risk cohorts to NYC may also account for plateauing mortality rates among younger individuals.[19] Over the past two decades, record numbers of foreign-born individuals have immigrated to NYC; nearly 2 million foreign-born residents were living in NYC in 1990, with 3 million foreign residents by 2008.[20] The cardiovascular health of foreign-born immigrants in the United States is not well established, but foreign-born women are reported to have higher mortality due to ischemic heart disease and stroke, and high rates of diabetes have been reported in a number of immigrant populations.[21]

### Race and sex differences in ischemic heart disease mortality trends

This is the first study to assess trends in IHD mortality by sex and race/ethnicity in young women and men in NYC. Young black women, but not young black men, were found to be at a higher IHD mortality risk than white men and women in the same 35–54 age group. Black women experienced the slowest rate of decline in IHD mortality among all women aged 35–54.

There are several potential explanations for these concerning findings. Black women have higher prevalence of cardiovascular disease risk factors, including obesity, hypertension, and diabetes, when compared to white women.[22–28] Black women also have higher mortality rates after presentation with acute coronary syndromes and lower long-term survival with IHD than whites, even after adjustment for differences in clinical characteristics and treatment.[29, 30][31] When black women present to medical attention with IHD, they tend to report fewer classic chest pain symptoms, regardless of the severity of CAD, and atypical symptoms at presentation are associated with poor outcomes.[32] In a recent analysis of 78 million ER visits for chest pain, black race and younger age were associated with lower rates of diagnostic test ordering and less triage to emergency status.[33] Poverty, stress, and other factors associated with race may contribute to the IHD risk observed in this vulnerable subgroup.[34–36] Therefore, interventions aimed at risk factor management and community and provider education about MI warning signs and risk among black women have the potential to favorably impact IHD mortality.

We recognize several limitations. First, race and ethnicity reported on death certificates could not be verified for accuracy. Race misclassification was unlikely to affect the designation of non-Hispanic white and black individuals, although ethnicity errors and census undercounting could affect both cohorts.[37] However, it is unlikely that such biases would systemically affect particular age, sex, or ethnic sub-groups. Second, relationships between age, sex and race/ethnicity and IHD mortality may represent competing risks for death from other causes such as trauma, potentially overestimating the risk of cardiovascular death in certain populations.[38, 39] In an analysis of large NHLBI databases, traditional Cox modeling and a competing risks model yielded divergent hazards of first IHD event in black men in comparison to whites.[39] Third, population growth that is not reflected in US census data may lead to inaccuracies in population estimates and mortality rates. Fourth, changes in the diagnosis of IHD and vital statistics reporting and the adoption of a newer diagnostic coding scheme, ICD-10, in 1999, could impact mortality trends. However, this coding scheme change does not affect the IHD group of diagnoses, which have a comparability ratio of 1.00.[40] Furthermore, nonparametric analysis of the points of change in mortality trends identified inflection points in the years before and after 1999 in several subgroups. Fifth, erroneous assignment of IHD as a cause of death may occur.[41, 42] Death certificate specificity for IHD diagnosis has been reported to be as low as 72%.[43] Even at autopsy, medical examiners occasionally attribute death to IHD in the absence of pathologic evidence of myocardial infarction, severe atherosclerotic coronary artery disease, or coronary thrombosis.[44] However, it is unlikely that such bias would systemically affect particular age, sex, or ethnic sub-groups. Finally, there is marked variation in IHD mortality in the US based on geography that is even greater among blacks.[6] Consequently, restriction of the analysis to NYC eliminates confounding by region and urbanization and permits study of the impact of race and sex on IHD mortality.[6]

## Conclusions

Despite improvements in medical therapy and aggressive interventions to treat IHD, the rate of decline in IHD mortality among younger individuals in NYC has slowed in recent years. IHD mortality trends in younger black women were particularly unfavorable and black women under age 55 are at increased risk of premature IHD death. Further investigation is warranted into targeted medical and public health interventions to reduce IHD mortality in this vulnerable population.
